# Development of a Theoretical Framework for Program Evaluation of the Impact of Policy, Systems, and Environmental Interventions on Individual‐Level Behavior

**DOI:** 10.1111/jhn.70307

**Published:** 2026-07-12

**Authors:** Krystal L. Hodge, Caitlin Kownacki, Kaitlyn Streitmatter, Rebecca L. Hagedorn‐Hatfield, Karah Mantinan, Jennifer McCaffrey

**Affiliations:** ^1^ Department of Food Science and Human Nutrition University of Illinois Urbana Champaign Illinois USA; ^2^ Illinois Extension University of Illinois Urbana Champaign Illinois USA; ^3^ Altarum Institute Ann Arbor Michigan USA

**Keywords:** policy, systems, and environment interventions, program evaluation, PSE, theory of change mapping, theory‐based frameworks

## Abstract

**Background:**

As community nutrition education programs have expanded to include policy, systems, and environmental (PSE) initiatives to increase access to nutritious food and safe physical activity, the impact of these efforts on the consumption of healthier foods and physical activity levels is not clear. Research shows the effectiveness of PSE changes on individual‐level behavior in controlled environments and in other public health disciplines. However, more evidence and guidance is needed to show the impact of PSE interventions in practice on individual‐level nutrition and physical activity behaviors. The PSE Intervention and Evaluation Framework (PSE‐IEF) was developed to explain the process by which implemented PSE activities may result in behavior changes within the priority audience.

**Methods:**

Framework development involved a review and Theory of Change mapping process of a list of PSE changes used in the Supplemental Nutrition Assistance Program‐ Education (SNAP‐Ed) Program, an environmental literature scan to identify relevant theories and related factors, and an expert review. The environmental scan examined how PSE changes are conceptualized as drivers of behavior change and how their impacts are measured at the individual level. Sources were identified through database searches, grey literature review, and citation tracking, with inclusion limited to U.S.‐based, English‐language documents published in the past 10 years. Twelve frameworks or theories were reviewed for their focus on individual behavior, ability to capture behavior change over time, and recognition of individual choice in when or whether change occurs. The PSE changes theory of change mapping results were reviewed for alignment with the selected framework and applied to a logic model for PSE implementers. Forty‐five practitioners reviewed the framework for relevance, comprehensiveness, and comprehensibility, with revisions made iteratively until no substantial changes to structure or content were received.

**Results:**

The final PSE‐IEF describes how PSE programs are implemented at the organizational level and how they might impact the target audience at the individual level. To see outcomes at the individual level, organizational level activities must be completed, including staff awareness, engagement, action and maintenance activities to produce PSE outputs available for target audience use. Precaution Adoption Process Model stages of awareness, engagement, decision, action, and maintenance describe the responses at both the organizational and individual level over time.

**Conclusions:**

The resultant framework can be used to guide PSE intervention and evaluation in community‐based programs and to increase the evidence base illustrating the impact of PSE interventions on individual behavior and health.

## Summary

- Program evaluations of policy, systems, and environment (PSE) interventions to increase access to nutritious food and safe physical activity are often limited to measuring reach within populations without fully demonstrating the program's impact on the health behaviors or health outcomes of the target audience.
- Practitioners planning and implementing these PSE interventions must consider assessing the impact of these interventions on individual‐level health behavior. This will provide needed evidence of the impact and importance of these interventions for program funders and other stakeholders.

## 1 Introduction

Encouraging healthy eating and physical activity to reduce disease risks is a longstanding priority in the United States (U.S.). Nutrition education to promote healthy eating and physical activity emphasizes increasing knowledge, improving skills, and changing attitudes. These efforts may include one‐on‐one nutrition therapy, group education sessions, and technology‐based interventions. While many interventions are successful, impact can be limited if the surrounding environment does not support healthy dietary and physical activity behaviors [1].

Nutrition education programs funded by the U.S. Department of Agriculture (USDA) like the Supplemental Nutrition Assistance Program‐Education (SNAP‐Ed) expanded in 2012 to include comprehensive public health approaches to address policy, systems, and environmental (PSE) limitations preventing audiences from accessing nutritious foods and safe physical activity opportunities [2, 3]. For over three decades, SNAP‐Ed played a significant role in providing nutrition education programming to SNAP‐eligible audiences across the U.S [4]. Through partnerships and collaborations, the program incorporated direct nutrition education, PSE interventions, and the strategic use of social marketing to impact dietary and physical activity behaviors [4].

The use of PSE changes with nutrition education is grounded in the Social Ecological Model (SEM) and behavioral economics [5, 6]. The SEM is a well‐known theory that describes how an individual is influenced by their surrounding systems and environments [7]. These include the microsystem, or immediate setting of the individual; the mesosystem, or settings like the home and family, school, and peers; exosystem or informal and formal settings such as the neighborhood and community, media, and social networks; and macrosystem which includes policies and societal norms. This systems‐based model has been applied to describe how environments impact behaviors, and to justify the need for interventions to improve those environments [8, 9]. For example, at the mesosystem level, school and workplace food environment initiatives and wellness policies have been shown to support healthy nutrition‐related behaviors in those settings [10, 11]. At the exosystem level, community gardens and farmers markets increase access to fruits and vegetables in those communities while policies at grocery stores and markets that support Electronic Benefit Transfer use increase access to food for SNAP recipients and are positively associated with diet quality [12, 13].

While the SEM focuses on how the environment affects one's choices, behavioral economics focuses on how choices are made. Behavioral economics is the study of how social, cognitive, and emotional factors influence people's decisions [14]. Traditional economic theory assumes people will choose their preferred option regardless of how the options are presented, but behavioral economics shows that people often select default options even when better alternatives exist. Default bias can encourage healthier decisions if they are the easiest or most automatic option [15]. PSE interventions apply behavioral economics by increasing the visibility of healthy choices through signage, labeling, and strategic product placement. These cues can influence selection and consumption decisions, which are difficult to change through sensory manipulation alone [15].

Behavioral economics has been widely applied in obesity prevention interventions, often alongside direct education [14, 15]. Techniques like altering defaults, modifying packaging, and using promotional strategies nudge individuals toward healthier choices while preserving their freedom to choose. These approaches are effective when embedded within broader systems that support sustained behavior change. Beyond the implementation of a PSE change, it is important to understand how a person becomes aware of the change, decides to or not to engage with it, and whether to use the new options, leading to increased access to healthy options, and changes in related behaviors.

A goal of nutrition education programs is sustainable healthy behavior changes that result in measurable impacts on health. Achieving health behavior change requires passive and active components. Passive components include PSE changes that create supportive conditions, while active components involve an individual's response to external PSE conditions. This dual approach is evident in many public health interventions [16]. For example, enacting a seatbelt law reduces the risk of mortality, but individuals must still choose to wear their seatbelts. Therefore, when implementing PSE changes to improve nutrition and physical activity, the behavioral aspects that guide individuals toward adopting and sustaining healthier behaviors must still be considered. While these active and passive strategies are critical elements of behavior change, SEM and behavioral economics do not specify the pathway to that behavior change. The documentation and evaluation of individual level behavior changes resulting from nutrition and physical activity‐related PSEs is limited. Evaluation of PSE impact in the past has focused on quantifying the number of initiatives implemented and the potential number of people who have access to the implemented initiative without directly quantifying how the initiative is changing the behavior and health of the target audience [17, 18, 19]. To date, only one other conceptual framework for PSE has been proposed. While the individual plus PSE (I + PSE) framework provides valuable insights into the role of PSE changes in public health in conjunction with direct education, it does not fully capture the specific pathways by which PSE interventions lead to individual‐level behavior change [20].

The purpose of this paper is to present a theoretical framework, the PSE Intervention and Evaluation Framework (PSE‐IEF), to conceptualize how PSE changes can lead to changes in dietary and physical activity behaviors. The PSE‐IEF can be used to plan and implement evaluations to ensure appropriate metrics are collected to demonstrate how PSE changes lead to specified behavior changes, and to understand underlying mechanisms when interventions do not achieve intended outcomes. This framework will add to the literature by introducing a model for measuring the impact of PSE activities on individual level outcomes, and will ultimately be accompanied by a series of materials to support practitioners' implementation and evaluation plans.

## 2 Materials and Methods

### 2.1 Framework Development Process

A multifaceted approach was used to develop the PSE‐IEF. Initially, the authors conducted a review of the SNAP‐Ed PSE changes listed in the National Program Evaluation and Reporting System (N‐PEARS), a required platform used by program implementers to report national SNAP‐Ed programming activity [21]. Following this, an environmental scan was conducted to examine behavioral theories and frameworks related to behavior change in PSE‐related interventions. Results of the SNAP‐Ed PSE changes review and environmental scan informed the development of an initial framework. The preliminary PSE‐IEF underwent several rounds of expert evaluation and refinement, culminating in the final version presented in this manuscript.

### 2.2 SNAP‐Ed PSE Changes Adopted Theory of Change Mapping

The SNAP‐Ed N‐PEARS reporting system had a list of nutrition, food access, and physical activity‐related PSE changes that SNAP‐Ed programs could report [22]. This list included potential nutrition or physical activity‐related PSE changes implemented by SNAP‐Ed programs nationally [22]. A team of SNAP‐Ed program leaders, evaluators, trainers, and faculty with over 70 years of combined experience in nutrition education, dietetics, and program implementation, independently reviewed the PSE changes list using a theory of change approach to logically describe how a new PSE change could influence a person's behavior. Each person drafted a pathway of behavior change for each item, met to identify emerging patterns, and came to a consensus on behavior change pathways for each listed PSE change. For example, if the PSE change was for a market to process SNAP for purchases, people with SNAP benefits must be aware of the change. The shopping location has to be of interest to the person and they must make a decision to shop or not shop at that market. For that PSE change to result in a change in health, there needs to be consistent access to healthy food at the market, consistent use of the market by the target audience, selection of the healthy items, and continued consumption of those healthy foods. Two examples of the Theory of Change Mapping Process are illustrated in Table 1.

**Table 1:** Theory of change mapping process examples.

Context	PSE change	Result of PSE change	Individual steps mapped for behavior change					
Nutrition	Healthy food and beverage defaults	Availability of healthy foods and drinks	Become aware of healthy options	Perceive the healthy options as the better choice	Select the healthy options	Consume the healthy options	Continue to select and consume the healthy options	Improve biometrics
Physical Activity	Complete streets environmental change	Availability of safe streets for physical activity	Become aware of safe streets	Perceive the streets are safe	Be motivated to begin using the streets	Continue to use the streets	Increase physical activity levels	Improve biometrics

### 2.3 Environmental Scan and Framework Selection

Next, an environmental scan was conducted to identify existing frameworks used to describe how PSE changes lead to behavior change, and to learn how researchers have measured the impact of PSE implementation. The research questions for the scan were:

1. What research and guidance are available on measuring individual behavior change outcomes of PSE interventions in public health, nutrition, and physical activity settings?
2. What theories or frameworks are available for evaluating behavior change among participants in PSE‐related public health, nutrition, and physical activity interventions?
3. What factors influence progression towards behavior changes in public health, nutrition, and physical activity interventions?

The environmental scan methodology was developed by the team of authors with oversight by a senior author who has over 18 years of experience providing research and evaluation services, including environmental scans and literature reviews, for organizations working on food and nutrition, physical activity, and other public health topics. The search criteria were tested to ensure they were appropriately identifying research in line with the research questions prior to starting the search. The environmental scan was conducted from November to December 2024 and included searches in PubMed and Google Scholar using search strings shown in Table 2. A title/abstract filter was applied for the search. A grey literature (e.g., government reports, industry documents) search was conducted in Google using the same search strings with the first 20 outputs of each search being reviewed. Articles were limited to those published since 2014 and available in English. Reviewed articles addressed one or more of the research questions and were PSE focused, including topics from public health interventions beyond physical activity and nutrition (e.g., smoking cessation, vaccination programs) due to the recognized limitation in the availability of PSE‐related evaluation tools for nutrition and physical activity‐related PSE interventions and the successful implementation and measurement of PSE approaches in these other public health spaces [17, 23]. The environmental scan produced 500 articles with additional articles reviewed during snowball search methods of PSE‐related topics (e.g., seatbelt policy). Data were extracted from articles related to factors influencing behavior change (e.g., social media, motivation), PSE components (e.g., policy change, environmental interventions), behavioral focus (e.g., nutrition, physical activity), and guiding theory or framework. The scan results provided background on health behavior change and informed the selection of the guiding framework and influencers that aligned with the PSE change mapping results as described below.

**Table 2:** Search strategy used for PubMed and google scholar to identify policy, systems, and environmental (PSE) related frameworks and evaluation techniques.

Research question	Search terms and Boolean/Phrase	Articles(*n*)
What research and guidance are available on measuring individual behavior change outcomes of PSE interventions in public health, nutrition, and physical activity settings?	(PSE OR “Policy, system*, and environment*” OR “policy intervention*”OR “environment* intervention*” OR “policy change*” OR “environment* change*” OR “system change”) AND (“behavior change” OR behavior) AND (outcome* OR evaluation* OR measure*) AND (nutrition OR “public health” OR “physical activity”)	52
What theories or frameworks are available for evaluating behavior change among participants in PSE‐related public health, nutrition, and physical activity interventions?	(Framework OR theor* OR model) AND (“behavior change” OR behavior) AND (nutrition OR “public health” OR “physical activity”) AND (“PSE” OR “Policy, system*, and environment*” OR policy OR environment* OR system*)	388
What factors influence progression towards behavior changes in public health, nutrition, and physical activity interventions?	(“Stage* of Change” OR “Process* of Change”) AND (“behavior change” OR behavior OR influence) AND (nutrition OR “public health” OR “physical activity”) AND (“PSE” OR “policy system environment*“ OR policy OR environment* OR system*)	60

*Note:* Using * at the end of a search phrase allows various, unknown characters to follow.

#### 2.3.1 Literature Guiding Framework Selection

**Figure 1:**
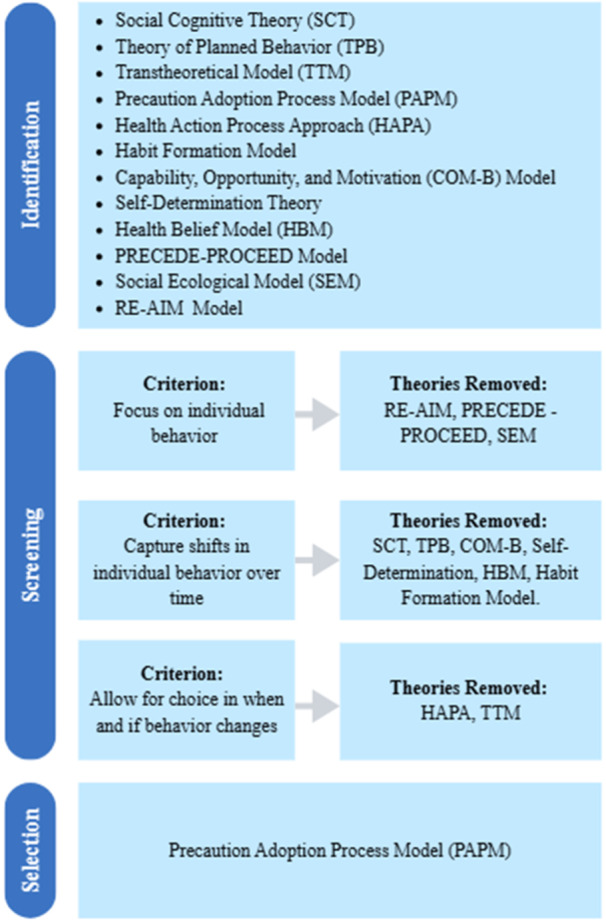
Flowchart of theories evaluated and screening criterion for final theory selection.

The environmental scan identified 12 theories or frameworks for consideration. The four most predominant theories for consideration were the Social Cognitive Theory (SCT), Theory of Planned Behavior (TPB), Transtheoretical Model (TTM), and Precaution Adoption Process Model (PAPM). Other theories identified in the literature on a less frequent basis included the Health Action Process Approach (HAPA); Habit Formation Model; Capability, Opportunity, and Motivation (COM‐B) Model; Self‐Determination Theory; Health Belief Model (HBM); PRECEDE‐PROCEED Model; Social Ecological Model (SEM); and RE‐AIM (reach, effectiveness, adoption, implementation, maintenance) Model. Background on each theory type was explored for alignment with the steps identified in the PSE mapping process. Three guiding criteria for framework selection were implemented by the project team: (1) does the theory focus on individual behavior, (2) can the theory capture shifts in individual behavior over time and (3) does the theory allow for choice in when, and if, they change behavior. The pathway to the final model is shown in Figure 1.

Planning and implementation theories (e.g., PRECEDE‐PROCEED, RE‐AIM) and multi‐level or ecological models (e.g., SEM) were excluded from consideration in relation to the first guiding criteria due to the lack of focus solely on individual behavior change. The remaining theories that can capture change in individual behavior are often grouped into one of two theory groups: continuum models or stage models [24, 25] and were reviewed as such. Continuum models aim to identify predictive factors like knowledge, self‐efficacy, and attitudes that influence the likelihood of behavior change as part of a gradual process [25]. Examples include the SCT and TPB which posit that the probability of health behavior change increases as more theoretical constructs are satisfied. Behavior progresses along a continuum, with change occurring once all relevant constructs are met. The environmental scan identified some connection between continuum models and how PSE changes might impact individual behavior. The SCT emphasizes environmental factors as influencers on behavioral change, suggesting that adjustments like offering healthier food choices in schools can model and reinforce positive habits [26]. The TPB focuses on subjective norms and perceived behavioral control, implying that policies and systems which make healthy choices easier, such as subsidies for fruits and vegetables, can boost perceived control and alter societal expectations [27]. However, these theories were not selected to guide the PSE‐IEF due to their inability to capture the shift in behavior, as they more often capture intent compared to behavior [25]. In addition, these models likely fail to capture variation in individual readiness to engage in behavior change. As such, the SCT, TPB, COM‐B, Self‐Determination, HBM, and the Habit Formation Model were removed from consideration.

In contrast to continuum models, stage models propose that behavior change occurs as part of a dynamic process where individuals progress through a series of distinct stages, each with specific predictors that drive an individual towards behavior change [24, 28]. Examples include the TTM, HAPA, and the PAPM. In these models, stages reflect a person's level of engagement with the desired behavioral outcome. Specific theoretical constructs must be addressed to facilitate progression to the next stage, ultimately leading to the adoption and maintenance of the health behavior change.

As PSE interventions seek to invoke change among groups of people likely at different stages of behavior change, utilization of a stage model is appropriate. Use of stage models in population‐based smoking cessation efforts have demonstrated that these models can provide a successful framework to maximize engagement with larger populations [29]. Additionally, stage models often consider many of the constructs of continuum models, like self‐efficacy, just at varying stages of readiness for change [30, 31]. For example, the HAPA includes a motivation (pre‐intentional) phase and a volition (post‐intentional) phase making it a stage model, but explicitly incorporates continuum‐style constructs (e.g., self‐efficacy, risk perception) within the stages. However, with only two phases, the intricate details of behavior change across time make it less intuitive for evaluators seeking to understand movement across the behavior change spectrum.

The TTM is a commonly referenced stage model of behavior change across various sectors of public health [29, 32, 33]. The TTM includes five stages of change: precontemplation, contemplation, preparation, action, and maintenance [32]. Additionally, the TTM considers constructs that influence the shift between stages including the process of change, decisional balance, and self‐efficacy [32]. While the TTM has been explored with policy and environmental changes in smoking cessation for population health change, nutrition and physical activity literature has explored the use of TTM in direct education interventions through randomized control trials or pre‐post study designs [34, 35, 36, 37]. As such, little is known about the TTM to guide individual behavior change as a result of PSE interventions that might or might not be associated with direct education. In addition, the TTM lacks stages to describe an individual's decision to not move to the next stage. This is paramount for PSE interventions as personal choice to engage and act upon the PSE is essential.

An alternative to the TTM is the PAPM which is based on the premise that people change behavior as a result of understanding the intent and benefit of engaging with the intervention [38]. Research on the PAPM is sparse compared to the TTM but has increased in recent years. In 2006, it was noted that only 11 publications were available through a PubMed search on PAPM [39], but our own recent search identified over 100 articles published on this framework. Similar to TTM, the PAPM has been applied to multiple public health interventions including smoking cessation, cancer screening, home radon testing, and osteoporosis prevention, where people take deliberate actions to reduce health risks [24, 39]. The PAPM has been applied to understand decisions in policy and environmental interventions including lightning safety policies in schools [40] and guided the development of frameworks to understand engagement in prevention behaviors such as prenatal screenings [41], positioning it to guide the development of a framework for other PSE interventions.

The PAPM comprises seven stages of change: unaware, unengaged, undecided, decided not to act, decided to act, action, maintenance [42]. Unlike the TTM, PAPM includes the decision to not engage or act upon behavior change, despite being aware of the opportunity to engage in behavior change activities. In addition, although the naming of the stages may appear similar, the context and definition of the stages are distinct. While the TTM provides time‐bound definitions (e.g., intending to take action in the next 6 months), the PAPM describes the cognitive and psychological factors likely at play in determining which behavior change stage a person is in, and their likelihood of shifting to the next stage [28]. Given these additional factors and the alignment of the PAPM stages with the steps identified during the theory of change mapping process, the PAPM was selected to inform the PSE‐IEF.

### 2.4 Model Development

The PSE changes list theory of change mapping results were aligned with the PAPM stages to explore how individual‐level behavior would shift at each stage of the PAPM. The resulting PSE‐IEF is an application of the PAPM to include the program development and implementation aspects, and the change process following PSE implementation. The PAPM model for PSE implementation and evaluation was then applied to a logic model structure to visually explain how and why the parts of a program work together to achieve outcomes. Logic models are a recognizable structure for PSE implementers and have been suggested to help visualize how PSE change is part of a larger strategy to improve health [43]. In the PSE‐IEF, the logic model outlines the steps involved in implementing a PSE change and the PAPM explains how individuals become aware of these changes and decide to engage (or not engage) in behavior change that leads to improved health outcomes. Lastly, commonly identified influencing factors from the environmental scan were applied to the PSE‐IEF to demonstrate external factors that might influence behavior change.

### 2.5 Initial Content Validity and Expert Feedback

The initial draft of the PSE‐IEF underwent multiple rounds of expert review and testing from individuals in various roles of PSE implementation and evaluation. Qualitative assessment of content and face validity was conducted, an approach that has been applied to the development of other frameworks and evaluation tools in the public health space [44, 45, 46, 47]. Content validity refers to how well a framework captures the constructs it is intended to represent [47]. This is often evaluated through qualitative interviews with people who have relevant experience or expertise, focusing on whether the framework is comprehensive and relevant. Face validity, a type of content validity, involves activities like cognitive interviews where the target audience tests the framework and shares feedback [47]. This process helps determine if the framework's components are clear and understandable in practice.

The authors created a list of interview questions to assess relevance, comprehensiveness, and comprehensibility of the framework, using published literature to guide the development of the interview guide (48). Five face validity questions assessed understandability (e.g., Do the word choices make it easy for different groups of people such as practitioners, community members, or researchers to use this framework?), and clarity (e.g., Does the model offer a clear framework for evaluating program effectiveness?). Nine content validity questions assessed relevance (e.g., How well does the tool account for the complexities of PSE community work?), practical application (e.g., How do you see yourself using this in your work?) and scope (e.g., Does the tool adequately address short, medium, and long‐term outcomes?). In addition, three general questions on strengthening the PSE‐IEF were assessed, including what would you change or add to strengthen the tool, do you have recommendations for making the model more inclusive or adaptable, and how does the model support continuous improvement of community programs?

Altogether, 45 practitioners, each with 2 or more years of experience implementing nutrition and physical activity focused PSE initiatives, provided feedback throughout the development process including four in‐person meetings, four virtual meetings, and multiple email communications and one‐on‐one meetings. Framework revisions occurred iteratively with additional feedback collected after changes were made. Suggested changes were related to the content (e.g., add stages for staff implementation) and structure (e.g., clarify flow of arrows and movement within framework). Feedback was gathered until no new substantial changes to the structure or content were received.

## 3 Results

**Figure 2:**
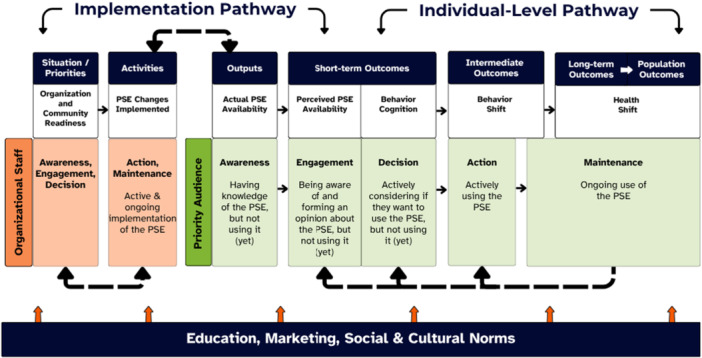
The policy, system, and environmental (PSE) intervention and evaluation framework.

The final PSE‐IEF (Figure 2) establishes a basis for planning and implementing PSE change interventions and evaluations. A logic model structure and the PAPM describe how organizations implement PSE changes and how individuals may react to those changes, including their awareness, decision‐making process, and adoption of behaviors that influence health outcomes.

### 3.1 Description of PSE Intervention and Evaluation Framework

#### 3.1.1 Organizational Staff

Awareness, Engagement, and Decision: The PSE‐IEF begins on the left side with the Implementation Pathway and the organizational staff's awareness, engagement, and decision‐making, situated within the organization's situation and priorities. “Staff” may include paid employees or unpaid volunteers. In this stage, the PSE implementation plan is being developed and there is a formal or informal assessment of readiness within the organization and community, including a determination of whether the PSE implementers are aware of, engaged, and understand the purpose of the PSE, and have made the decision to support implementation.

Action, Maintenance: Next is the organizational staff's action and maintenance, described as the “active and ongoing implementation of the PSE.” In this phase, PSE changes are implemented and maintained by staff making the PSE available for priority audiences to access and influence behaviors.

#### 3.1.2 Priority Audience

Awareness: The first stage of the PAPM is “unaware” in which an individual has not considered behavior change. Before PSE implementation, the priority audience is considered unaware as the PSE output is not yet available. Awareness begins to occur during the “output” phase of the Implementation Pathway where there is an actual change in availability. It is at this point “awareness” can be assessed. Awareness occurs as individuals begin to learn of the PSE and its intended purpose but have not yet formed an opinion about it. It is described in the PSE‐IEF as “having knowledge of the PSE, but not using it (yet).” Once individuals are aware and move to the next stage of engagement, there is no backwards movement to awareness.

Engagement: The second stage of the PAPM is “unengaged” in which individuals are aware of the PSE and forming an opinion about it. In the PSE‐IEF, engagement occurs as a short‐term outcome where the priority audience's perception of the PSE's availability is considered. At this stage, individuals are aware of and forming an opinion about the PSE but not using it yet. The opinion is informed by internal (e.g., past experiences, beliefs, knowledge, and skills) and external (e.g., community norms, media, nutrition education) factors that influence whether they perceive the PSE as accessible and useful to them. These opinions are what moves (or not) the individual from the engagement to decision‐making stage. Those who move forward in the PAPM can always revert to opinion forming in the engagement phase.

Decision: The third stage of the PAPM is “undecided” in which individuals have formed their opinion but not decided to act. In the PSE‐IEF, transition is made here to the Individual‐Level Pathway where decision also occurs as a short‐term outcome as behavioral cognition of intent and benefits of using the PSE are developed. This is described as “actively considering if they want to use the PSE, but not using it (yet)”. From here, individuals move to the fourth stage (decided not to act) or fifth stage (decided to act). In this way, the PAPM makes clear that the decision not to act is a choice distinct from someone still considering. These decisions are influenced by constructs of continuum behavior change theories like self‐efficacy, social norms, or beliefs about perceived susceptibility, among others. Factors that influence decision to act include risk perceptions, positive attitudes, strong subjective norms, positive modeling, high self‐efficacy, and pros of acting [39]. These same factors can apply to the PSE‐IEF, where individuals feel motivated (e.g., positive attitude) to engage with the PSE and choose to use it in a manner that is appropriate and accepted by their peers.

Action: The sixth stage of the PAPM is “acting” where individuals begin using the PSE. In the Individual‐Level Pathway, action occurs as an intermediate outcome where individual behaviors start to shift because of the PSE. In the PSE‐IEF, this step is described as an individual “actively using the PSE”. Acting can be influenced by constructs of other theories including simple cues to action that nudge individuals towards PSE use, access to the resources needed to act (e.g., having a bike to ride on a new bike path) or understanding the PSE change and being confident in one's ability to use it (e.g., ability to interpret what sodium labels on a menu mean).

Maintenance: The final stage of the PAPM is “maintenance” in which an individual sustains a new behavior, assimilating the action into their norms. In the PSE‐IEF, maintenance is the “ongoing use of the PSE” and begins as an intermediate outcome where repeated behavioral shifts are developing into a habit. Through ongoing use of the PSE and associated behavioral shifts, long‐term health and population outcomes can be achieved that result from behavior changes. Throughout this process, it is important to note a person can revert to a previous stage if they choose not to maintain an action.

Education, Marketing, Social & Cultural Norms: Direct education activities, social marketing, and social and cultural norms impact how participants learn about the PSE, potentially influencing opinions and motivation to use PSE. The impact of these on the decision‐making process will vary depending on the individual's exposure and social and cultural context. An example of the PSE‐IEF applied to a physical activity‐related example is presented in Supplemental Figure 1. Supplemental Figure 2 and 3 illustrate supporting tools developed as part of a toolkit (not included) to support practitioners with evaluation at each stage of the PSE‐IEF.

## 4 Discussion

The PSE‐IEF is a theoretical framework that describes how an individual takes action on an implemented PSE that can lead toward health improvement. Evaluation of PSE interventions have utilized frameworks in the past like the CDC's *Framework for Program Evaluation in Public Health* (49) or the RE‐AIM framework (50), but the majority of the literature on these focuses on evaluating the implementation process and outputs (e.g., change in foods available) but lacks focus on individual outcomes (e.g., change in awareness or actual dietary intake). A 2024 scoping review of evaluation tools on Early Childhood PSE Change, including the RE‐AIM framework, found that of the 36 studies utilizing PSE‐focused evaluation tools, zero focused on attitudes, behaviors, or health outcome metrics [17]. Despite documented implementation and reach of PSE interventions, there is insufficient documentation of individual‐level and population‐level outcome changes within the PSE evaluation literature [2].

The PSE‐IEF can be used to plan program evaluations to assess the effect of PSE changes on individuals' behaviors and health outcomes. For example, PSE interventions are increasingly popular to address food and nutrition security challenges, involving collaborations with community stakeholders including healthcare, state and local government, and academia (51). Food as Medicine is one area where the PSE‐IEF can support evaluation (52,53). Healthcare organizations are increasingly utilizing community‐based food programs for the prevention and management of diet‐related conditions. These interventions seek to improve nutrition security by enhancing access to nutritious foods and addressing food access challenges through approaches like produce prescriptions or medically tailored meals or groceries (54). As these initiatives are adopted, it is important to assess individual‐level impacts resulting from policy and systems level changes. The PSE‐IEF can also support practitioners as they gauge how a person decides to act upon the increased access to food provided through these initiatives.

Although the PSE‐IEF was initially developed through a nutrition and physical activity lens (i.e. SNAP‐Ed), a concerted effort was made to ensure its translation to other realms of public health with opportunities for testing and use. Efforts to improve policies, systems, and environments for better health will continue despite the elimination of SNAP‐Ed through the work of Expanded Food and Nutrition Education Program (EFNEP), Cooperative Extension, and other public health programs. Initiatives involving cross‐sector collaborations offer innovative approaches to improve public health across settings including childcare, schools, and healthcare, to name a few. Evaluation of individual behavior‐change in these initiatives will be vital to demonstrate effectiveness and advocate for sustainable funding. Utilization of this framework can also help dietitians and public health practitioners to strengthen their evaluation skills and understanding of individual‐level facilitators or obstacles related to their PSE interventions.

The PSE‐IEF has been reviewed by practitioners and offers several key strengths. First, the multi‐step development approach ensured that the framework was grounded in an evidence‐based theoretical foundation, while incorporating direct input from PSE experts. Second, the framework offers adaptability across various PSE interventions. Nonetheless, the qualitative content validity testing is just the first step and further testing is required to quantify content validity, providing stronger support for the PSE‐IEF's suitability and utilization, ensuring both validity and reliability. The next stage of testing is underway and will be published separately and presented in a forthcoming PSE‐IEF Toolkit. Other PSE implementers, including those previously in SNAP‐Ed and new PSE researchers, are invited to test the framework and associated tools for application to their work. Through the use of this framework, the goal is the development of new, PSE‐specific evaluation tools that help expand the evidence on the impact of PSE interventions.

## 5 Conclusion

The PSE‐IEF describes how PSE implementation can result in individual‐level behavior changes in priority audiences. It outlines PSE implementation plans and the PAPM based decision‐making process a person experiences while determining whether to use an implemented PSE. The framework can also be used as a communication tool with program implementers and stakeholders in the design of program evaluations. Nationally, significant gaps exist in the implementation of PSE initiatives due to the recent elimination of SNAP‐Ed program funding. As nutrition professionals, community organizations and Cooperative Extension programs continue to adjust, resources for training and supporting the advancement of this work are needed. This framework and forthcoming PSE‐IEF Toolkit will provide guidance to new PSE implementers and researchers to demonstrate impact beyond adoption.

## Author Contributions

Caitlin Kownacki and Jennifer McCaffrey identified the need for the framework. Rebecca L. Hagedorn‐Hatfield., Karah Mantinan, and Krystal L. Hodge conducted the literature scan. Krystal L. Hodge, Caitlin Kownacki, Kaitlyn Streitmatter, and Jennifer McCaffrey reviewed the SNAP‐Ed Changes Adopted list. All reviewed the literature results and Changes Adopted list review results. Rebecca L. Hagedorn‐Hatfield, Krystal L. Hodge, Caitlin Kownacki, and Kaitlyn Streitmatter selected the guiding theory for the framework. Krystal L. Hodge and Rebecca L. Hagedorn‐Hatfield developed the initial PSE‐IEF framework. All revised and edited the framework. All wrote and edited the manuscript.

## Conflicts of Interest

The authors declare no conflicts of interest.

## Supporting information


Supporting File

## Data Availability

Data sharing not applicable to this article as no datasets were generated or analysed during the current study.
