# Supplementary material for: Development of a Theoretical Framework for Program Evaluation of the Impact of Policy, Systems, and Environmental Interventions on Individual‐Level Behavior
Source: J Hum Nutr Diet. 2026 Jul 12;39(4):e70307. doi: 10.1111/jhn.70307 (PMC13358025; doi:10.1111/jhn.70307)

**Supplemental Material**

**Supplemental Figure 1** provides an example of the PSE Evaluation Framework applied to a physical activity-related example. In this example, consider you are working with a school to increase students’ and staff’s physical activity, and to support a local Active Streets Initiative which promotes safe, active transportation. After meeting with school leaders, parents, and students, your team develops a plan to add a bike rack at the school so students and staff have a place to safely store their bike. The plan includes selecting a safe, visible location, coordination with city officials for installation, and a promotional campaign of flyers, school social media, school announcements, and emails sent to parents. You also develop a plan for how the team will monitor staff and students’ response to the new bike rack. After installation, you follow up with your team to start implementing your monitoring plan to determine if the bike rack is being used and how it is impacting the people who are using it. The PSE Evaluation Framework shows the change in behavior and outcomes as individuals move through the PAPM stages.

Supplemental Figure 1: An Example of The Policy, System, and Environmental (PSE) Intervention and Evaluation Framework


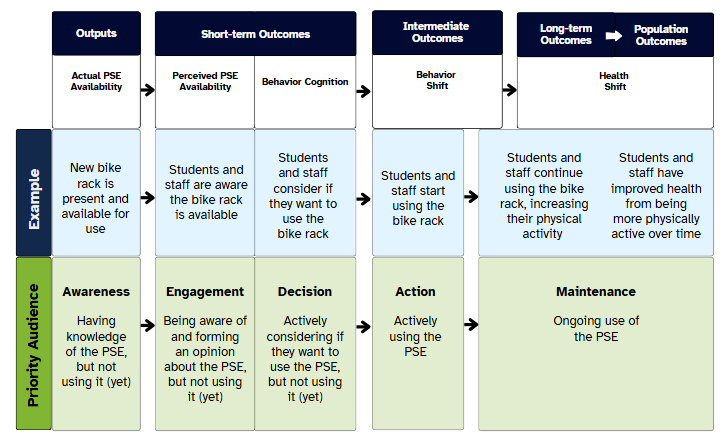


**Supplemental Figures 2 and 3** show supporting tools that were developed to support practitioners with evaluation at each stage of the PSE-IEF. The Decision Tree figures are intended to guide practitioners through identifying what stage of the PSE-IEF the target audience is at to evaluate at that stage and identify opportunities to move behavior to the next stage. The Decision Trees are accompanied by questions to help practitioners know what to ask at each step of the process and will be available in a forthcoming PSE-IEF Toolkit. Testing of the Decision Trees and questions is underway and will be published separately.

**Supplemental Figure 2: The Policy, System, and Environmental (PSE) Intervention and Evaluation Framework Decision Tree Implementation Pathway**

***
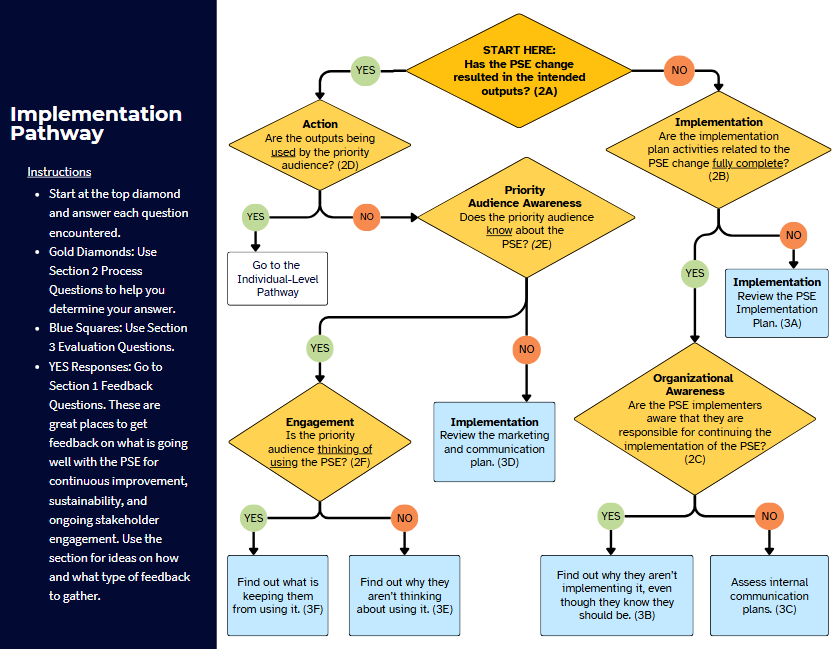
***

**Supplemental Figure 3: The Policy, System, and Environmental (PSE) Intervention and Evaluation Framework Decision Tree Individual Pathway**
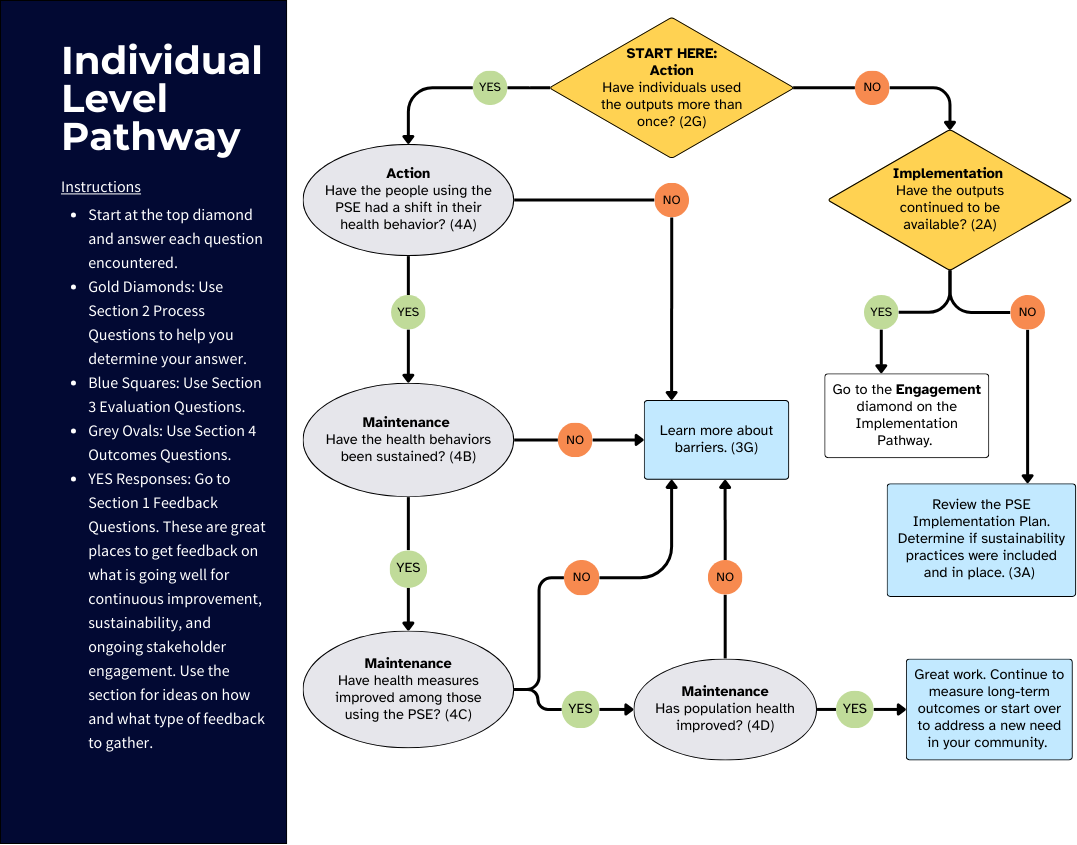

Supplement: Supplementary file 1 — Supporting File [file JHN-39-0-s001.docx]
